# Extending the utility of the WHO recommended assay for direct detection of enteroviruses from clinical specimen for resolving poliovirus co-infection

**DOI:** 10.1186/s13104-018-3155-6

**Published:** 2018-01-18

**Authors:** Temitope Oluwasegun Cephas Faleye, Moses Olubusuyi Adewumi, Naomi Princess Ozegbe, Oluwaseun Elijah Ogunsakin, Grace Ariyo, Faith Wuraola Adeshina, Oluwaseun Sarah Ogunga, Similoluwa Deborah Oluwadare, Johnson Adekunle Adeniji

**Affiliations:** 1 Department of Virology, College of Medicine, University of Ibadan, Ibadan, Oyo State Nigeria; 2 Department of Microbiology, Faculty of Science, Ekiti State University, Ado Ekiti, Ekiti State Nigeria; 3 WHO National Polio Laboratory, University of Ibadan, Ibadan, Oyo State Nigeria

**Keywords:** Enteroviruses, Polioviruses, WHO, Surveillance, Nigeria

## Abstract

**Objectives:**

In a polio-free world there might be reduced funding for poliovirus surveillance. There is therefore the need to ensure that enterovirologist globally, especially those outside the global polio laboratory network, can participate in poliovirus surveillance without neglecting their enterovirus type of interest. To accomplish this, assays are needed that allow such active participation.

**Results:**

In this study we describes a sensitive and specific utility extension of the recently recommended WHO RT-snPCR assay that enables independent detection of the three poliovirus types especially in cases of co-infection. More importantly, it piggy-backs on the first round PCR product of the WHO recommended assay and consequently ensures that enterovirologists interested in nonpolio enteroviruses can continue their investigations, and contribute significantly and specifically to poliovirus surveillance, by using the excess of their first round PCR product.

**Electronic supplementary material:**

The online version of this article (10.1186/s13104-018-3155-6) contains supplementary material, which is available to authorized users.

## Introduction

There are 13 species in the genus *Enterovirus* (EV) (family Picornaviridae, order Picornavirales). The Polioviruses (alongside other nonpolio enteroviruses [NPEVs]) belong to species C and are the prototype members of the genus. In 2015, the WHO included the reverse transcriptase-seminested polymerase chain reaction (RT-snPCR) assay described by Nix and colleagues [1] for direct (cell culture independent) detection of enteroviruses from clinical specimen [2]. The Nix et al. [1] assay is an upgrade (seminested and Consensus Degenerate Hybrid Oligonucleotide Primers [CODEHOP]) version of the Oberste et al. [3] assay. It has been shown that this algorithm is very sensitive for cell culture independent enterovirus detection and identification [4–6]. However, we [7] have recently shown that the assay lacks the capacity to resolve enterovirus types present in cases of co-infection. Significantly, the prevalence of enteroviruses co-infections in Nigeria is underscored by the independent emergence of 29 lineages of circulating Vaccine Derived Polioviruses (cVDPVs) between 2004 and 2014 [8], most of which are of recombinant origin [9]. This necessitates the need for assays that can detect and resolve enterovirus co-infections.

We had also shown [7] that by including primers 189 and 187 (subsequently referred to in this study as Species Resolution Primers) in the second round PCR of the WHO recommended RT-snPCR assay the resolving power of the assay could be improved. However, in a polio-free world, where there might be reduced funding for enterovirus surveillance, assays are needed that can in one swoop detect and resolve enterovirus co-infections, including different poliovirus types. Therefore, in this study, we extend the utility of the WHO recommended RT-snPCR assay by inclusion of three poliovirus-specific forward primers in the second round assay, thus, making it possible to independently detect the three poliovirus types even in cases of co-infection.

## Main text

**Fig. 1:**
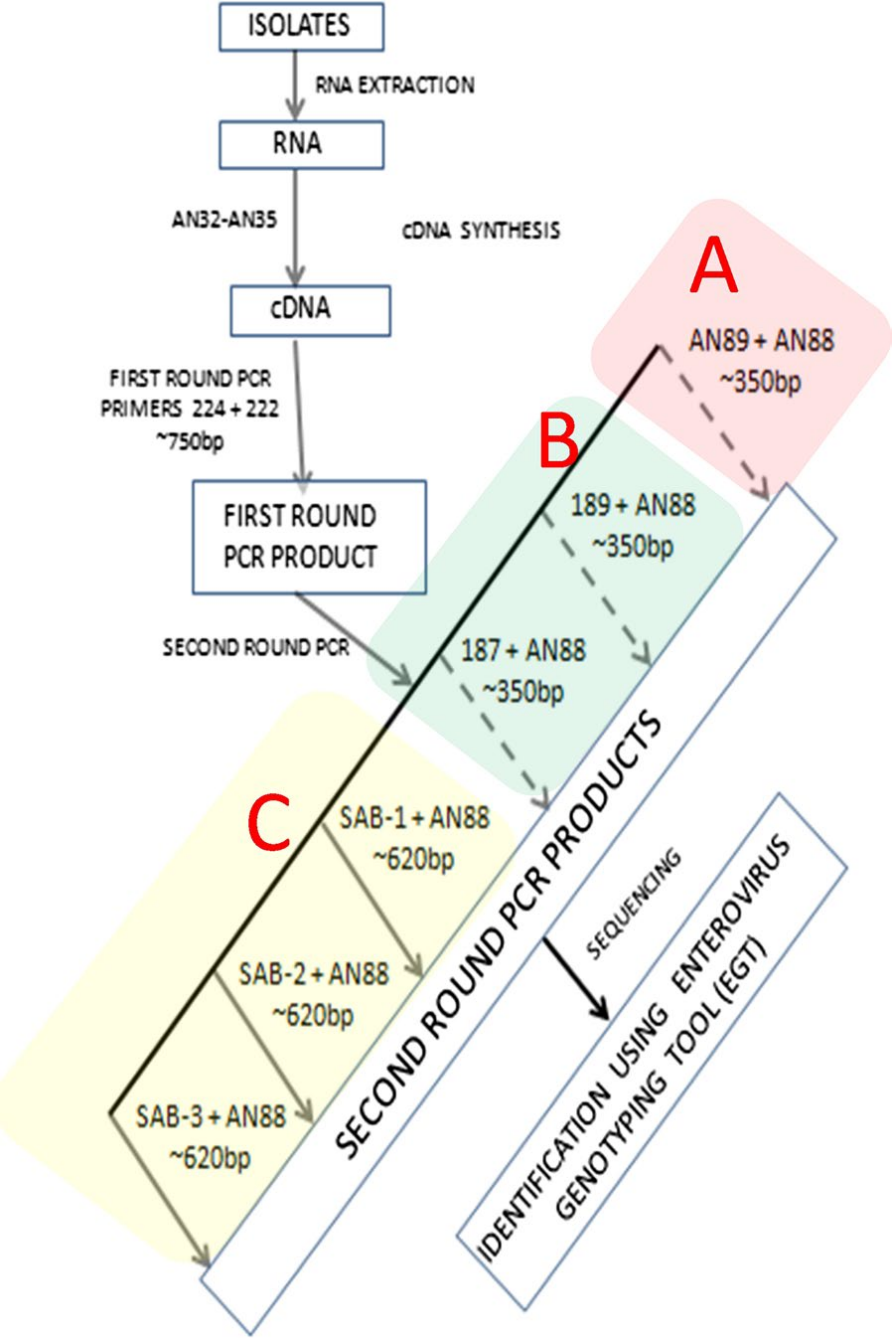
The algorithm followed in this study. A—The WHO 2015 recommended assay, B—Species Resolution Modification (SRM) and C—Poliovirus Resolution Modification (PRM)

Sixteen enterovirus isolates (Table 1) previously recovered from sewage contaminated water were used as reference samples in this study and analyzed as depicted in the study algorithm (Fig. 1). Isolation and characterization of samples 1 through 12 have been previously described [10]. Briefly, samples 1 through 8 are enterovirus species B (EV-B), and were isolated on RD cell line [10]. Samples 9 to 12 are enterovirus species C (EV-C) and were isolated on MCF-7 cell line [10]. Samples 13 to 15 are Sabin poliovirus 1–3 respectively. Samples 13 and 15 were isolated and characterized by the WHO Environmental Surveillance (ES) laboratory in Ibadan, Nigeria and provided to us as references for Sabin 1 and 3 polioviruses, respectively. Sample 14 (a poliovirus 2) was isolated as part of a previous study [11] but identified subsequent to the study. Sample 16 is a mixture of six isolates, two species B (samples 1 and 3) and four species C (samples 11, 13, 14 and 15) (Table 1).

**Table 1:** Identity of isolates determined using the algorithm

S/N	Information prior this study		Results of this study						
	Previously determined ID	Species	WHO 2015	Species Resolving Modification (SRM)		Poliovirus Resolving Modification (PRM)			Final summary of isolates identified
			AN89	189	187	Sab 1	Sab 2	Sab 3	
1	E3	EV-B	E3	unx	E3	Neg	Neg	Neg	E3
2	E19	EV-B	E7	unx	E19	Neg	Neg	Neg	E7/E19
3	E7	EV-B	E7	unx	E7	Neg	Neg	Neg	E7
4	E19	EV-B	Neg	E20	Neg	Neg	Neg	Neg	E20/E19
5	E7	EV-B	E7	Neg	E7	Neg	Neg	Neg	E7
6	E19	EV-B	Neg	unx	E19	Neg	Neg	Neg	E19
7	E7	EV-B	E7	unx	E7	Neg	Neg	Neg	E7
8	UNT	EV-B	E7	unx	E7	Neg	Neg	Neg	E7
9	CV-A13	EV-C	CV-A13	unx	CV-A13	Neg	Neg	Neg	CV-A13
10	CV-A13	EV-C	CV-A13	CV-A13	CV-A13	Neg	Neg	Neg	CV-A13
11	CV-A13	EV-C	CV-A13	CV-A13	unx	Neg	Neg	Neg	CV-A13
12	CV-A13	EV-C	CV-A13	CV-A13	Neg	Neg	Neg	Neg	CV-A13
13	PV-1	EV-C	PV-1	PV-1	PV-1	PV-1	PV-2	PV-3	PV-1, PV-2, PV-3
14	PV-2	EV-C	PV-2	PV-2	unx	Neg	PV-2^a^	unx	PV-2
15	PV-3	EV-C	PV-3	PV-3	unx	Neg	Neg	PV-3^b^	PV-3
16	PV-1, PV-2, PV-3, CV-A13 (S/N 11), E7 (S/N 3), E3 (S/N 1)	EV-B and C	PV-2	unx	PV-1	PV-1	PV-2^a^	PV-3^b^	PV-1, PV-2, PV-3

The primers included in the Species and Poliovirus Resolving Modifications (SRMs and PRMs) are referred to in the text as Species and Poliovirus Resolving Primers (SRPs and PRPs), respectively

The PRM did not amplify any of the isolates in samples 1–12

*E* Echovirus, *EV* enterovirus, *PV* Poliovirus, *CV* Coxsackievirus; *unx* unexploitable due to bad sequence data, *Unt* untypable, *Neg* negative (no amplification)

^a, b^Isolates are genetically the same

**Fig. 2:**
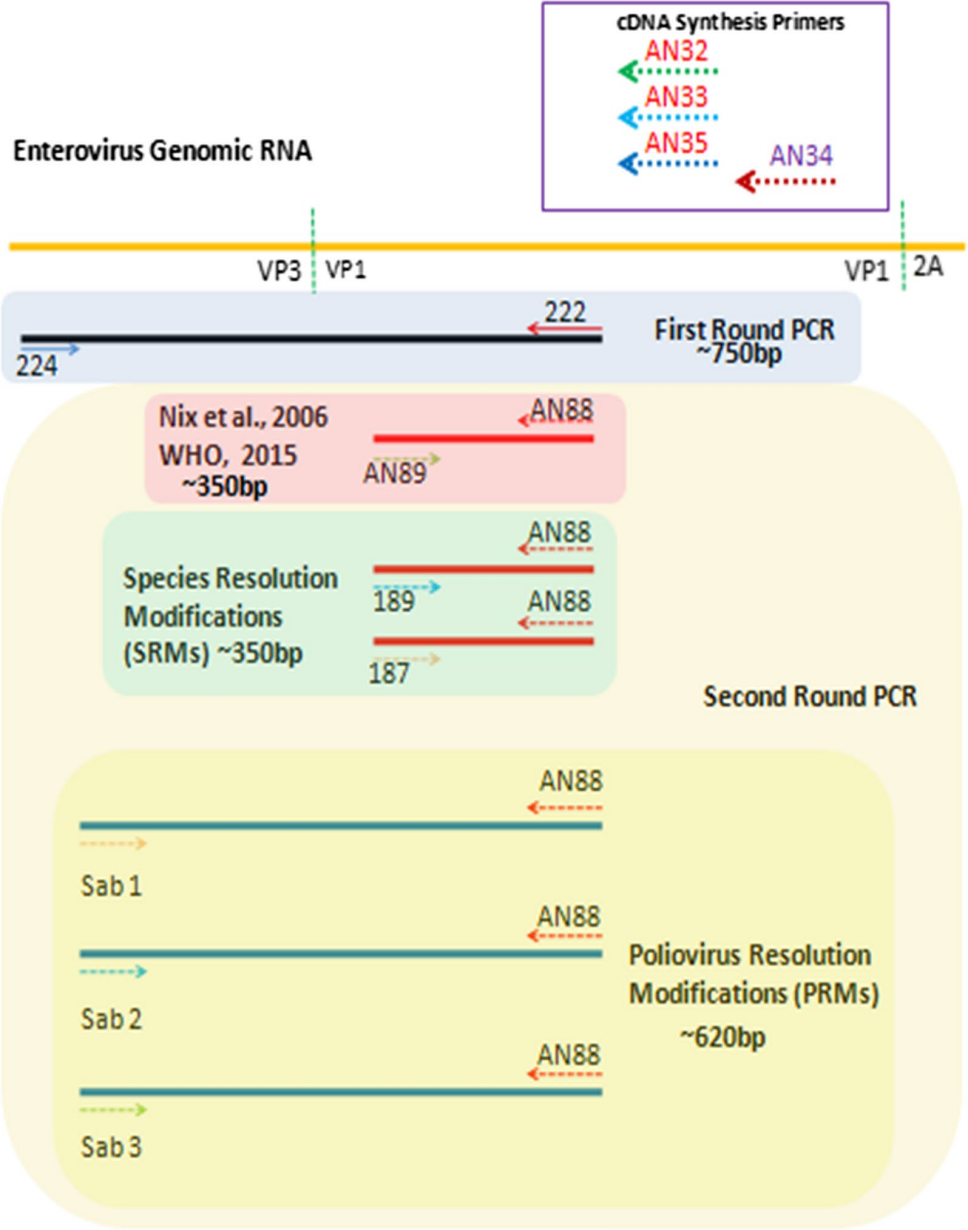
A schematic representation of the annealing sites of the different primers used in this study relative to the enterovirus genome and the consequent amplification product (arrows depict primers and their orientation)

All 16 samples were subjected to RNA extraction and RT-snPCR as previously described [12] but with slight modifications (Fig. 1). Precisely, instead of three second round PCR assays, there were six as depicted in Figs. 1 and 2. The forward primers included in the remaining three assays are listed in Additional file 1: Table S1. A more detailed description of the molecular detection algorithm is provided in Additional file 2. All amplicons generated from the six second round PCR assays were shipped to Macrogen Inc, Seoul, South Korea, for purification and sequencing. The primers used for each of the second round PCR assays (Fig. 2) were also used for sequencing, respectively. The identities of the sequenced isolates were determined using the enterovirus genotyping tool [13]. Sequences newly generated in this study have been deposited in GenBank. The accession numbers are KX856914-KX856921.

The results of this study are detailed in Table 1. For isolates one to eight (all previously known to be EV-Bs [10]), the previously described assays [12] (Table 1; assays WHO 2015 and SRM, respectively) identified the isolates to a large extent in accordance with their previously stated types [10]. It also identified the previously unidentified sample 8 as E7. Furthermore, it showed the presence of E20 in an isolate (sample 4) previously shown to be only E19. As expected, the Poliovirus Resolving Primers (PRPs [Sab 1, 2 and 3]) did not amplify any of the isolates in samples 1 to 8 (Table 1). For isolates nine to twelve which were all previously identified as CV-A13 (EV-C) [10], the assay as previously described [12], identified the isolates in accordance with their previously stated types [10]. The PRPs did not amplify any of the isolates in samples 9 to 12, as expected (Table 1).

For isolates 13 to 15 (previously identified as Sabin PV-1, 2 and 3 respectively), the assay as previously described [12], identified the isolates in accordance with their previously stated types. However, the PRPs resolved the isolates. The Sabin-1 primer only detected sample 13 and confirmed it to be PV-1. The Sabin-2 primer produced the expected amplicon size in both samples 13 and 14. The sequence data subsequently confirmed both to contain two different Sabin 2 viruses that are 99.65% similar (data not shown). In fact, the Sabin 2 in sample 13 has an Isoleusine (I) at position 143 of VP1 while that in sample 14 has an Asparagine (N) at the same position. The Sabin-3 primer also produced the expected amplicon size in both samples 13 and 15. The sequence data also confirmed both to contain two different Sabin-3 viruses that are 99.65% similar (data not shown). Hence, the assay as previously described [12] (i.e. primers AN89 and the SRPs [189 and 187]) did very well in identifying all three polioviruses. The PRPs (Sab1–Sab3) also confirmed the identity of the three samples. The PRPs however, further showed that sample 13, which was previously identified by the GPLN algorithm [14] as PV-1 and confirmed by primers AN89 and the SRPs as such, also contained PV-2 and PV-3 (Table 1).

For sample 16 which contained four EV-Cs (PV-1, PV-2, PV-3 and CV-A13), and two EV-Bs (E3 and E7), all the six primers produced their expected band sizes. Sequence data analysis however showed that while the amplicon from primer 189 produced unexploitable sequence data, primers AN89, 187, Sab-1, Sab-2 and Sab-3 detected PV-2, PV-1, PV-1, PV-2 and PV-3, respectively (Table 1). Otherwise stated, AN89 and the SRPs (primers 189 and 187) only detected PV-2 and PV-1 respectively from the mixture while the PRPs specifically detected all the three poliovirus serotypes. Importantly, the PV-2 and 3 detected in sample 16 were exactly those found in samples 14 and 15 respectively (Table 1).

In this study, by including poliovirus serotype specific forward primers [15] in the second round PCR of the WHO recommended RT-snPCR [2] assay, we were able to selectively and specifically detect and identify the different poliovirus serotypes. More importantly, this was accomplished in a situation where six different enterovirus types were present. The results of this study showed that in such instance, the RT-snPCR assay recommended by the WHO [2] detected only one of the six different enterovirus types present (Table 1; sample 16). It therefore confirmed that the WHO recommended RT-snPCR assay [2] might not be dependable for the resolution of enterovirus mixtures. The results of this study further showed that the WHO recommended RT-snPCR assay [2] can be modified or tailored as described in this study (for detection and identification of the polioviruses) to specifically detect other enterovirus types especially in cases of co-infection. Such serotype-specific modifications would be very valuable for low-income economies as it will broaden the surveillance capacity of enterovirologists in such settings with minimal increase in cost.

It is pertinent to note that this modification appears to be more sensitive for poliovirus detection and identification than both the WHO RT-snPCR algorithm [1, 2] and the current algorithm for poliovirus identification in use by the GPLN [14]. For example, while the GPLN algorithm [14] and the WHO RT-snPCR algorithm [2] identified sample 13 as PV-1, the modification described here showed that sample 13 contained PV-1, 2 and 3. More importantly, sequence comparison showed that the PV-2 and 3 in sample 13, and those in samples 14 and 15 were different (data not shown).

Consequent of this discovery, the initial result of this reference ES sample was retraced in the WHO polio lab at Ibadan Nigeria. It was then discovered that the results of all the inoculated cell culture flasks (five L20B and one RD) [16] showed that PV-1, 2 and 3 were isolated from the parent ES sample, though in different flasks (unpublished data). However, the PV isolate in the L20B flask from which sample 13 was aliquoted was previously identified as PV-1 by the current GPLN poliovirus detection and identification algorithm [14]. Altogether, this suggests that the isolate in sample 13, contained PV-1, 2 and 3 but PV-1 had a titre that is significantly higher than others. Consequently, it was the type detected in sample 13 both by the GPLN algorithm and primers AN89, 189 and 187 (Table 1; sample 13). Further buttressing the influence of PV-1 titre hypothesis is the fact that the PV-2 and 3 in sample 13 could not be detected in the sample 16 mixture. Rather it was the PV-2 and 3 in samples 14 and 15 that were detected in sample 16 (Table 1).

Considering that about 25,000 genomic equivalents are required for the current GPLN algorithm to detect the presence of PV-1 [17], this finding is not unexpected. Rather it suggests that while the genomic equivalents of PV-1 in sample 13 might be up to the required, those of PV-2 and PV-3 are below and account for the inability of the assay to detect both. This has implications for the polio eradication and endgame strategic plan 2013–2018 [18] and particularly the WHO global action plan (GAP III) for poliovirus containment and sequential withdrawal of the Sabin strains [19]. For example, in the course of Sabin PV-2 containment, isolates containing Sabin PV-2 but with titre below the detection limit of the GPLN assay for Sabin PV-2 detection might be missed. It is therefore suggested that for containment, all isolates that contain any of the poliovirus types should be handled as potentially containing the other two types. Furthermore, to reduce the misclassification of mixed isolates with low titre components and consequently enhance the containment programme, effort should be put into increasing the sensitivity of the assays in use by the GPLN and others; like that described in this study. In addition, effort should also be put into mainstreaming serotype-independent NextGen sequencing strategies recently described for the polioviruses [20] and other Species C members [21].

Though in this study; cell culture isolates were used, it is pertinent to note that we have successfully detected PV2 (unpublished data) directly in stool suspension from an AFP case that was missed by the current cell culture based algorithm [16] for poliovirus detection. In a polio-free world there might be reduced funding for poliovirus surveillance. In such potentially resource limited setting, the modification described in this study might be of value because it allows the enlistment of nonessential facilities without the capacity or infrastructure for cell culture to participate in poliovirus surveillance. Particularly, in such settings, enterovirologists interested in nonpolio enteroviruses can continue their investigations, and also contribute significantly and specifically to poliovirus surveillance, by using the excess of their first round PCR product.

## Limitations

The limit of detection of the modification described here is currently not known. Hence, effort is ongoing to conduct spiking experiments with plaque purified and titrated reference isolates in a bid to better define the sensitivity of the assay. Furthermore, it is crucial to mention that the sequence data this modification provides do not cover the entire VP1 region. As such, unlike the ECRA assay [17], the sequence data generated might not be sufficient for extensive molecular epidemiology. Consequently, this modification is currently proposed as addenda and not as substitute for either the current GPLN algorithm [6] or the other cell culture independent assays [17, 22] with the capacity to provide sequence data of the entire VP1 region.

## Additional files

**Additional file 1: Table S1.** Sequences of the different forward primers used for second round PCR in this study.

**Additional file 2.** This supplement is a more detailed description of the methods used in this study.

## Abbreviations

AFP: acute flaccid paralysis
PCR: polymerase chain reaction
RT-snPCR: reverse transcriptase-seminested polymerase chain reaction
RNA: ribonucleic acid
cDNA: complementary deoxyribonucleic acid
RD: human rhabdomyosarcoma
UTR: untranslated region
VP1: virus protein 1
EV: Enterovirus
E: Echovirus
CV: Coxsackievirus
WHA: World Health Assembly
GPEI: Global Polio Eradication Initiative
OPV: oral polio vaccine
IPV: inactivated polio vaccine
ES: Environmental Surveillance
GPLN: global polio laboratory network
NPEV: nonpolio enterovirus
L20B: genetically engineered mouse cell line expressing the human cell surface receptor for poliovirus
WHO: World Health Organization
CPE: cytopathic effect
PE: PanEnterovirus
F: forward
R: reverse
MCF-7: breast cancer cell line
