# Supplementary material for: Extending the utility of the WHO recommended assay for direct detection of enteroviruses from clinical specimen for resolving poliovirus co-infection
Source: BMC Res Notes. 2018 Jan 18;11:47. doi: 10.1186/s13104-018-3155-6 (PMC5774100; doi:10.1186/s13104-018-3155-6)
Supplement: Supplementary file 1 — Additional file 1: Table S1. Sequences of the different forward primers used for second round PCR in this study. [file 13104_2018_3155_MOESM1_ESM.doc]

Table S1: Sequences of the different forward primers used for second round PCR in this study.

| **S/N** | **PRIMER NAME** | **PRIMER SEQUENCE** | **PRIMER BINDING REGION** | **REFERENCE** |
| --- | --- | --- | --- | --- |
| 1 | AN89 | CCAGCACTGACAGCAGYNGARAYNGG | VP1 | Nix et al., 2006 |
| 2 | 189 | CARGCIGCIGARACIGGNGC | VP1 | Oberste et al., 2003 |
| 3 | 187 | ACIGCIGYIGARACIGGNCA | VP1 | Oberste et al., 2003 |
| 4 | Sab 1 | AGTCGTCCCTCTTTCGACA | VP3 | Sadeuh-Mba et al., 2013 |
| 5 | Sab 2 | TAGGGTTGTTGTCCCGTTG | VP3 | Sadeuh-Mba et al., 2013 |
| 6 | Sab 3 | TGTGGTGCCACTGTCCACC | VP3 | Sadeuh-Mba et al., 2013 |

**Note:** Sab 1-Sab 3 was originally designed by M.L. Joffret but first detailed in Sadeuh-Mba et al., 2013 as unpublished data
