# Supplementary material for: Extending the utility of the WHO recommended assay for direct detection of enteroviruses from clinical specimen for resolving poliovirus co-infection
Source: BMC Res Notes. 2018 Jan 18;11:47. doi: 10.1186/s13104-018-3155-6 (PMC5774100; doi:10.1186/s13104-018-3155-6)
Supplement: Supplementary file 2 — Additional file 2. This supplement is a more detailed description of the methods used in this study. [file 13104_2018_3155_MOESM2_ESM.docx]

**ADDITIONAL METHODOLOGY**

***RNA Extraction and cDNA synthesis***

In accordance with the manufacturer’s instructions, all samples were subjected to RNA extraction and cDNA synthesis using JenaBioscience RNA extraction kit and Script cDNA synthesis kit (Jena Bioscience, Jena, Germany) respectively. Primers AN32, AN33, AN34 and AN35 [1] were used in combination for cDNA synthesis.

***Seminested Polymerase Chain Reaction (snPCR) Assay for Enterovirus VP1 gene***

All primers were re-constituted in concentrations of 100μM and first round PCR was done in 50μL reactions. The first round PCR contained 0.5μL each of primers 224 and 222 [1], 10μL of Red load Taq, 10μL of cDNA and 29μL of RNase free water. A Veriti thermal cycler (Applied Biosystems, California, USA) was used for thermal cycling as follows; 94°C for 3 minutes followed by 45 cycles of 94°C for 30 seconds, 42°C for 30 seconds and 60°C for 60 seconds with ramp of 40% from 42°C to 60°C. This was then followed by 72°C for 7 minutes and held at 4°C till terminated.

The second round PCR was done in 30μL reactions. It contained 0.3μL each of forward and reverse primers (Figure 2), 6μL of Red load Taq, 5μL of first round PCR product and 18.4μL of RNase free water. As shown in the algorithm (Figure 1), six different second round PCR assays were done using the first round PCR product as template. Hence, all six second round PCR assays used different forward primers (Supplementary Table 1) but the same reverse primer (AN88) (Figure 2). Based on the expected amplicon size (Figure 2), the PCR extension time for the second round assays were 30 seconds for primers AN89, 189 and 187 [1, 2, 3] and 60 seconds for primers Sab-1, Sab-2 and Sab-3 [15]. The extension temperature was however retained at 60^0^C. Subsequently, PCR products were resolved on 2% agarose gel stained with ethidium bromide, and viewed using a UV transilluminator.

***Nucleotide sequencing and enterovirus identification***

All amplicons generated from the six second round PCR assays were shipped to Macrogen Inc, Seoul, South Korea, for purification and sequencing. The primers used for each of the second round PCR assays (Figure 2) were also used for sequencing, respectively. The identities of the sequenced isolates were determined using the enterovirus genotyping tool [13].

Note: References and Figures are available in the full text
